# Microtopography and Seasonal Soil Moisture Govern Methylmercury Dynamics in Upland Soils of a Subtropical Coastal Montane Watershed

**DOI:** 10.1021/acsomega.6c06624

**Published:** 2026-09-24

**Authors:** Xizi Yang, Martin Tsz-Ki Tsui, Zengchen Yu, Chenzi Yang, Mengwei Yuan, Shaoyi Wang, Ganxing Liu, Luming Yang, Lin Liu

**Affiliations:** † School of Life Sciences, The Chinese University of Hong Kong, Shatin, New Territories, Hong Kong SAR 999077, China; ‡ Department of Earth and Environmental Sciences,The Chinese University of Hong Kong, Shatin, New Territories, Hong Kong SAR 999077, China

## Abstract

Vegetated landscapes are widely known as important sinks of mercury (Hg) but the controls on the production of neurotoxic methylmercury (MeHg) in terrestrial upland ecosystems remain unresolved, particularly at fine spatial scales. In this study, we investigated the distribution of total Hg (THg) and MeHg in surface soils across an elevational gradient in a subtropical coastal montane watershed separately in dry and wet seasons at Hong Kong of southern China. Mean surface soil THg was the highest in the lower-elevation forest zone and at lower levels in the middle- and upper-elevation shrubland/grassland zones. Topsoil (0–4 cm) MeHg increased from dry to wet season at lower-elevation forest sites and decreased or changed little at most upper-elevation sites, although site-level elevation-zone contrasts were not statistically significant after false-discovery-rate adjustment. At the site level (*n* = 11), the best-supported model within a 12-model candidate set for seasonal topsoil MeHg change included seasonal moisture change, plan curvature, and inorganic Hg (IHg = THg – MeHg; adjusted *R* ^2^ = 0.758, *p* = 0.0043), implying exploratory watershed-scale associations among moisture, microtopography, Hg substrate, and MeHg heterogeneity.

## Graphical abstract

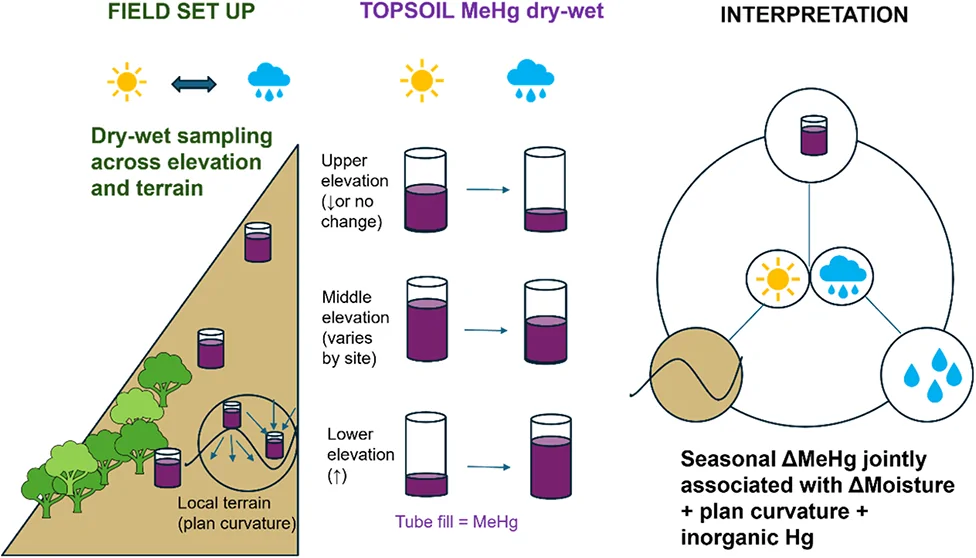

## Introduction

Mercury (Hg) is widely regarded as a global pollutant as Hg can be emitted as gaseous elemental Hg [Hg(0)] and transported over long distances in the atmosphere before deposition onto water and land. Vegetated landscapes accumulate atmospheric Hg mainly through foliar uptake of Hg(0) and additionally through wet and dry deposition of the inorganic form [Hg­(II)] formed by oxidation of Hg(0).^1^ However, the largest concern stems from the microbial conversion of deposited Hg­(II) to organic, highly toxic methylmercury (MeHg) by a suite of predominantly anaerobic microbes possessing the two-gene cluster *hgcAB*, including sulfate-reducing bacteria, iron-reducing bacteria, methanogens, fermenters, and other less-characterized clades.^2–4^ Metagenomic evidence also indicates putative *hgcAB*-carrying lineages with terminal oxidases that may support oxygen tolerance.^5^ MeHg can extensively bioaccumulate and biomagnify in natural food webs, often leading to high levels in top predators.^6^

In the last 4–5 decades, aquatic ecosystems such as lakes, wetlands, and coasts have been widely examined with respect to Hg cycling, especially MeHg production, because of the concern of elevated human uptake of highly toxic MeHg through the consumption of fish and seafood products.^6^ A recent synthesis reviews Hg cycling across atmospheric, terrestrial, and aquatic compartments.^7^ In contrast, we know relatively little regarding the controls of environmental factors on mediating MeHg production and its spatial distribution in upland environments, where there can be a complex interplay of elevation, topography, vegetation cover, and hydrology. Boreal forest studies have examined how forest harvest and hillslope redox conditions affect MeHg formation and export.^8,9^ Recent food web studies showed strong bioaccumulation and efficient biomagnification of MeHg in upland ecosystems.^10^

From a few studies, it is known that soil MeHg can be derived from litterfall^11^ and/or direct precipitation,^12^ but the predominant source of MeHg is believed to be derived from in situ microbial methylation within the organic horizons on the terrestrial landscape.^13^ Upland ecosystems are known as direct sources of MeHg to downstream aquatic ecosystems through hydrological transport,^14^ and empirical evidence suggested that aqueous MeHg is bound with dissolved organic matter as an intact complex mobilized from organic horizons.^15^ Thus, organic horizons of surface soil can serve as biogeochemical hotspots of MeHg production in upland ecosystems.

Currently, China is the largest global emitter of anthropogenic Hg mainly through coal combustion and smelting,^16^ even though there has been a consistent decline in the last 10–15 years.^17^ Hong Kong, a coastal metropolis of southern China, has been demonstrated to receive a large outflow of atmospheric Hg pollution from the Chinese mainland, especially the Pearl River Delta of Guangdong Province.^18^ At coastal sites, Hg(0) can be oxidized through the mixing of coastal winds with oxidants such as bromine.

Thus, this study aimed to assess soil THg levels and their variation along an elevational gradient, which also leads to a gradient of vegetation covers,^19^ in an upland landscape of Hong Kong. We also examined how soil MeHg varied spatially and seasonally because terrestrial organic soils can be an entry point for MeHg into food webs.^10,20^ Specifically, we investigated elevation, season, vegetation type, and topographic attributes as potential factors influencing THg and MeHg concentrations in soils at different depths.^21–25^ We also used the percentage of THg as MeHg (i.e., % MeHg) as a proxy for net Hg methylation potential and to identify potential biogeochemical hotspots along the elevation gradient.^26^ Previous studies have identified spatial MeHg production hotspots in peatlands^26^ and characterized boreal wetlands as MeHg sources or sinks in relation to soil acidity, C/N ratio, and small-scale flooding.^27^

## Materials and Methods

### Study Sites and Sample Collection

This study was conducted within a 3.1 km^2^ catchment area located among Tai Mo Shan Country Park (at a higher elevation) and Shing Mun Country Park (at a lower elevation), two adjacent country parks in the central part of Hong Kong, China (Figure 1a–c). Tai Mo Shan Country Park spans approximately 200–957 m a.s.l., whereas Shing Mun Country Park spans approximately 80–647 m a.s.l. The Tai Mo Shan Country Park covers an area of 14.4 km^2^ around Tai Mo Shan where the highest peak (957 m a.s.l.) exists in Hong Kong, while the Shing Mun Country Park commands a site of 14.0 km^2^. Shing Mun Reservoir, built for urban water supply needs in 1930s, collects water from different catchments including our study sites along a channel called Tai Shing Stream. Both country parks are protected, undeveloped upland with no buildings, roads, or impervious surfaces within or upslope of the sampling plots, and surface drainage is organized along Tai Shing Stream and its tributaries. Sites were chosen a priori to span elevation, vegetation type, and topographic position (ridge, slope, valley); the ridge/valley grouping of each site (by the topographic wetness index (TWI); see Data Analysis) is given in Table S1. The study area generally features a humid subtropical climate. However, there is a slight difference in climate among our sampling sites. Temperature is in general lower (17.2–18.0 °C), while rainfall is higher (1991–3030 mm) at the summit (>900 m a.s.l.) than those in the lowland (22.0–23.0 °C; 1780–2680 mm) based on Hong Kong Observatory records (2014–2024 normals); study-year (2024) monthly values for the summit (Tai Mo Shan) and lowland (Tsuen Wan) stations are shown in Figure 1.

**Figure 1:**
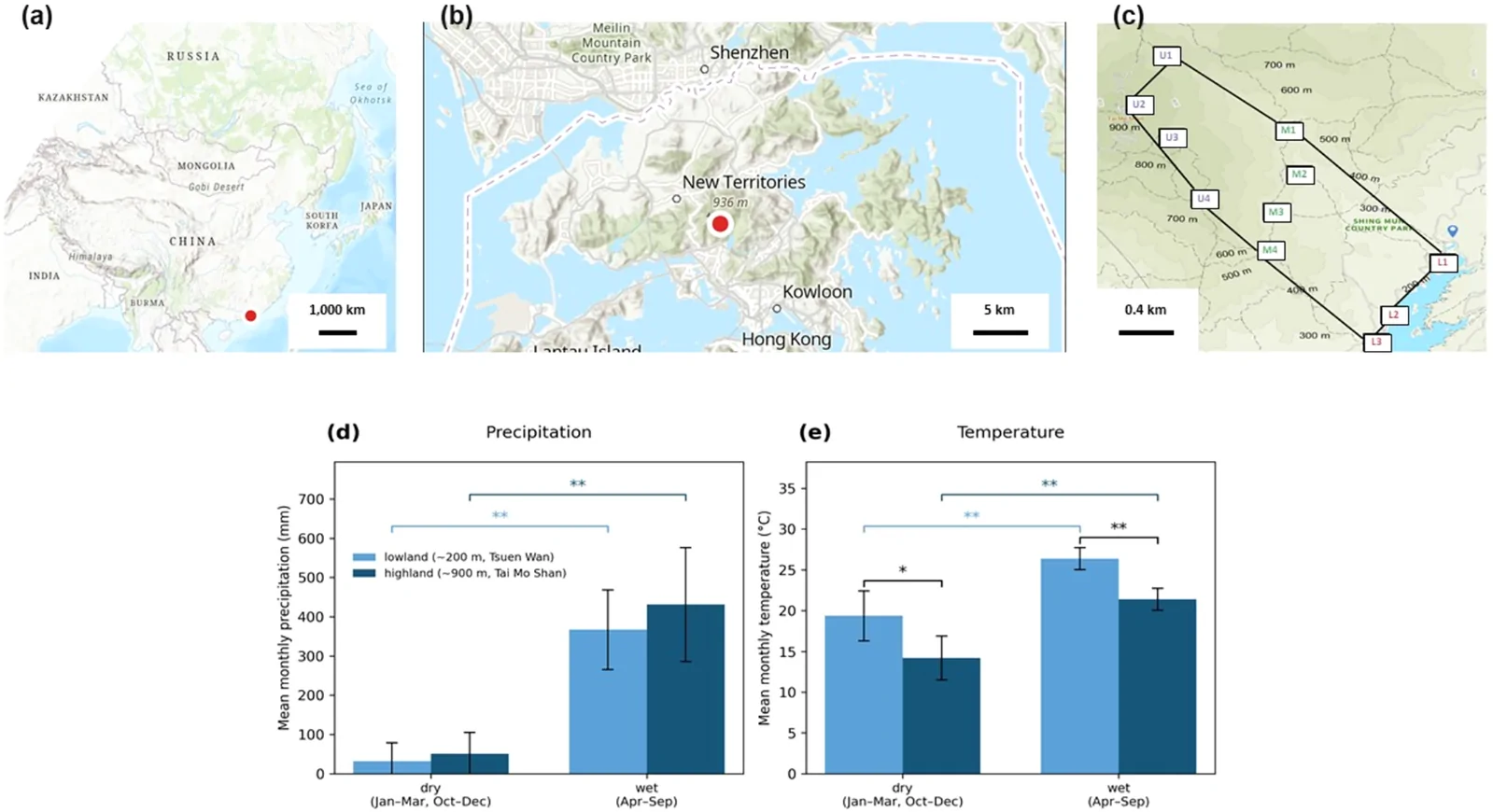
Location and climatic setting of the study area. (a) Location of Hong Kong SAR within China. (b) Location of the study area within Hong Kong. (c) Elevation/contour map showing the 11 sampling sites—lower (L), middle (M), and upper (U) elevation—along two sampling gradients; site-label colors indicate the elevation zone (red, lower; green, middle; purple, upper), consistent with Figures 2 and 3. (d) Mean monthly precipitation and (e) mean monthly temperature at the town of Tsuen Wan (lower-elevation) and Tai Mo Shan (upper-elevation) reference stations during the dry and wet seasons in 2024. Error bars are ±1 SD; *p < 0.05 and **p < 0.01. Climatic data source: Hong Kong Observatory. Panels (a) and (b) were generated using Esri basemaps. Panel (c) was generated using GeoInfo Map, Lands Department, Hong Kong SAR Government.

Two field sampling campaigns were conducted in 2024: 31 March, 5 April, and 10 April in the dry-season campaign, and 23 August, 28 August, and 27 September in the wet-season campaign. The 11 sampling sites extended from 200 to 910 m a.s.l. (Figure 1; Table S1), with vegetation transitioning from broadleaf forest at lower-elevation sites (L1–L3) to shrubland and grassland at middle-elevation sites (M1–M4) and upper-elevation sites (U1–U4) (Figure S1). During our study period, monthly precipitation was significantly higher (*p* < 0.05) during the wet (summer) season, while precipitation was slightly higher, without a statistically significant difference, at the upper elevation compared with the lower elevation (Figure 1d). In contrast, mean temperature was significantly lower at the upper elevation than at the lower elevation in both the dry season (winter) and wet season (summer) (*p* < 0.05) (Figure 1e).

In this work, we divided the sampling sites into three zones: lower elevation (3 sites at 200–218 m a.s.l.; L1, L2, L3), middle elevation (4 sites at 381–532 m a.s.l.; M1, M2, M3, M4), and upper elevation (4 sites at 742–910 m a.s.l.; U1, U2, U3, U4). At each sampling site, we identified a representative plot of 5 m × 5 m in which we collected triplicate 20 cm long soil core samples using a clean stainless steel auger. The three cores characterized within-plot variability, and the site was the independent unit for watershed-scale comparisons and seasonal-change models. Cores were sited on relatively gentle microrelief, avoiding tree bases (as required by park regulations) and exposed bedrock; where the auger could not reach 20 cm depth, the location was shifted and recorded. Sampling targeted rain-free conditions (little or no rainfall on the preceding day and a dry forecast); per-date on-day and previous-day rainfall are listed in Table S2, and a brief shower occurred during sampling on 31 March 2024. We divided each soil core into three sections based on the depth, i.e., 0–4, 4–10, and 10–20 cm.

All samples were transported on ice to the analytical laboratory within 4–6 h and subsequently stored in a −80 °C freezer until further processing. Individual soil samples (*n* = 198) were lyophilized by a Labconco benchtop freeze-dryer until constant weight and subsequently sieved through a 2 mm mesh housed in a stainless steel sieve that was carefully cleaned and dried between samples; sieving also homogenized each sample prior to subsampling.^28^ All processed samples were stored dry in the room temperature prior to analyses (see below).

### Soil Characterization and Mercury Analyses

Loss-on-ignition (LOI), a proxy for soil organic matter content, was determined for all soil samples through heating >5 g of subsamples in ceramic cups in a muffle furnace (Vulcan model 3-130) at 500 °C for 4 h,^29^ while soil moisture content was estimated as weight loss recorded during freeze-drying compared to the initial weight.

All processed soil and vegetation samples were determined for THg using a direct thermal decomposition-gold amalgamation method by NIC MA-3000 Direct Mercury Analyzer. Briefly, ∼40 mg of sample was weighed into a precleaned ceramic boat and placed into an autosampler of NIC MA-3000. For each batch of sample analysis, we included a blank and a standard reference material (MESS-4, Marine Sediment, THg reference value 0.09 ± 0.04 μg g^–1^, National Research Council Canada). Blank measurement always indicated negligible THg, with empty-boat blank readings of 0.006–0.01 ng Hg (about 1/100 of the signal of the lowest-concentration samples), while the recovery of THg (and standard deviation (SD)) of MESS-4 averaged at 90.9 ± 5.0% (*n* = 21). Our established detection limit was 0.75 ng g^–1^ dry wt and all samples had THg well above the detection limit.

All soil samples were extracted for MeHg through an alkaline-based method.^28,30^ Briefly, ∼300 mg of sieved soil sample was weighed into an acid-cleaned Teflon centrifuge vial (Oak-Ridge style, Nalgene), and 2 mL of 25% KOH–methanol (w/v) was added to the sample.^28^ The sample slurry was then heated and agitated at 70 °C in a shaking water bath for 4 h. Afterward, the sample slurry was centrifuged at 4000 rpm, and 100 μL of the supernatant was pipetted to a clean borosilicate glass vial with low-Hg deionized water with acetate buffer, and the pH of the solution was ∼4.9.^28^ To analyze MeHg, we added 50 μL of 1% ice-cold sodium tetraethylborate and the mixture was mixed continuously for 30 min. Analysis of MeHg was conducted using a Tekran Model 2700 Automated Methyl Mercury Analysis System following Wang et al.^28^ The calibration curve for MeHg analysis was developed using a MeHg standard solution (1 ng mL^–1^) obtained from Brooks Rand Instruments. In each analytical batch, we included a reagent blank and a standard reference materials (i.e., IAEA-456, Marine Sediment, certified at 0.125 ± 0.019 ng g^–1^, International Atomic Energy Agency). The mean recovery of MeHg (and SD) of IAEA-456 was 101 ± 11% (*n* = 10), and our method detection limit for MeHg was established at 0.04 ng g^–1^ dry wt, and most soil samples (196 out of 198) had MeHg above this detection limit. The two measurements below the method detection limit (0.027 and 0.028 ng g^–1^) were retained as measured in all data analyses.

### Topographic Characterization of Study Sites

The Topographic Position Index (TPI) and Topographic Wetness Index (TWI) are two important topographic attributes for characterizing landform morphology, modeling hydrological processes, and assessing ecological and environmental conditions that can be derived from a digital elevation model (DEM). TPI is widely used to quantify slope positions and support automated landform classification,^31^ while TWI is a common index used in hydrological analysis.^32^ In this work, we first imported the 30 m Copernicus DEM^33^ into the System for Automated Geoscientific Analysis (SAGA) tool in QGIS.^34^ Using the Compound Basic Terrian Analysis module, we derived primary topographic parameters including slope, aspect, plan and profile curvature, and TWI. Plan curvature is the curvature perpendicular to the slope (negative = concave/water-converging, positive = convex/water-diverging); profile curvature is the curvature along the slope; aspect is the downslope compass direction; TPI is the difference between a site’s elevation and the mean of its surroundings (negative = lower-lying than the surrounding terrain, positive = higher-lying); and TWI = ln­(specific catchment area/tan slope), a steady-state index of soil wetness used here to group sites into drier ridge and wetter valley positions. The TPI tool in the Morphometry module was then applied to compute TPI,^35^ and TWI calculation follows

$$TWI=\ln\left(\frac{A_{s}}{\tan\beta }\right)$$

*A*_s_ = specific catchment area, and β = local slope in the steepest downslope direction of the terrain in degrees.

### Seasonal-Change Modeling

The seasonal-change analysis used one dry-to-wet difference per site (*n* = 11). We evaluated a restricted set of 12 candidate models: an intercept-only model; 4 single-predictor models; 5 noncollinear two-predictor models; and 2 three-predictor models containing plan curvature and mean topsoil inorganic Hg (i.e., IHg = THg – MeHg) with either seasonal moisture change (ΔMoisture) or elevation. Separate model families were used for ΔMoisture and elevation because they were strongly correlated (*r* = −0.894). IHg removed MeHg from the substrate predictor; substituting THg was retained for a sensitivity analysis. All 12 ordinary-least-squares models were fitted in Python using statsmodels. Models were ranked by the Akaike Information Criterion corrected for a small sample size (AICc); ΔAICc, Akaike weights, adjusted *R* ^2^, and *F*-test *p* values are reported. AICc was calculated as −2 log *L* + 2*K* + 2*K*(*K* + 1)/(*n* – *K* – 1), with *K* including the regression coefficients and residual variance.^36^ The best-supported model was also evaluated with HC3 standard errors, variance-inflation factors, Cook’s distance, leverage, and leave-one-site-out refitting.

### Data Analysis

To avoid treating repeated depth layers as independent observations, replicate-level MeHg was analyzed with a maximum-likelihood linear mixed model of log­(MeHg), with random intercepts for site and physical core; each core was unique within one site and season. Depth and season were fixed factors, and the standardized continuous predictors were log­(IHg), LOI, moisture, and TWI. The mixed model used 191 complete observations from 66 physical cores at 11 sites; 6 observations lacked moisture and 1 lacked LOI. We report marginal and conditional *R* ^2^, leave-one-site-out sensitivity, and a THg substitution analysis (Table S6).^37^ A separate model with the same random effects evaluated log­(THg) as a function of standardized LOI with depth and season as covariates and used 197 observations because 1 LOI value was unavailable; sensitivity analyses tested an LOI × depth interaction and decomposed LOI into within-site and among-site components. Spearman correlations and two-sided Mann–Whitney *U* tests for elevation-zone and ridge-valley contrasts used one independent mean per site. Benjamini–Hochberg correction was applied within each season to the seven exploratory correlations and across the elevation-zone contrast family; ridge-valley *p* values are reported unadjusted. The percentage of THg present as MeHg (% MeHg) was calculated for each core from its season-specific MeHg and THg and then averaged across the three cores. Figure 2a displays the two-season site mean of THg; season-specific values were used for % MeHg, and two-season mean IHg (or THg in the sensitivity analysis) was used in the seasonal-change models. Sites were classified as ridge (TWI ≈ 4.8–5.9) or valley (TWI ≈ 7.3–9.7) following the natural break in TWI. Analyses used Python 3.13, statsmodels 0.14.6, and SciPy 1.17.1.^38^

**Figure 2:**
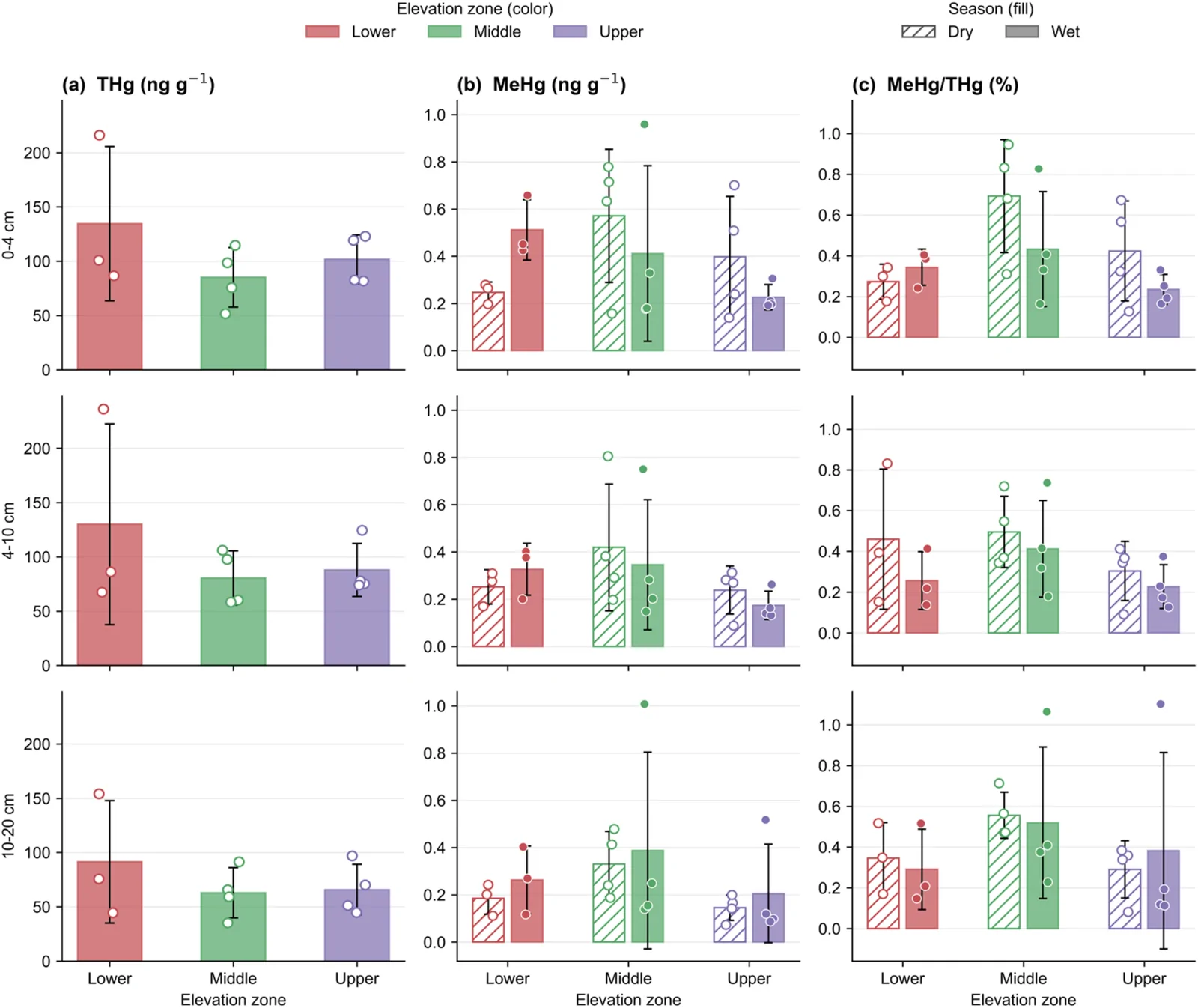
Site-level elevational patterns of soil (a) THg, (b) MeHg, and (c) % MeHg at 0–4, 4–10, and 10–20 cm, respectively. Bars are elevation-zone means calculated from one independent mean per site, error bars are ±1 SD among sites, and overlaid points show the individual site means. The bar color indicates the elevation zone (red, lower; green, middle; purple, upper). For MeHg and % MeHg, dry and wet seasons are distinguished by left/right position and hatched/solid fill, respectively. THg is displayed as the two-season site mean. No significance symbols are shown because no elevation-zone contrast remained significant after Benjamini–Hochberg adjustment across the elevation-zone comparisons.

## Results

### Influence of Elevation on Soil Mercury

Across all 198 observations, THg averaged 91.1 ± 50.5 ng g^–1^ (median: 81.4 ng g^–1^; range: 7.29–317.3 ng g^–1^) and MeHg averaged 0.315 ± 0.242 ng g^–1^ (median: 0.238 ng g^–1^; range: 0.027–1.317ng g^–1^). Mean topsoil THg was the highest at the lower elevation (134.5 ng g^–1^), lower at the middle elevation (85.1 ng g^–1^), and intermediate at the upper elevation (101.7 ng g^–1^) (Figure 2a; Tables S3–S5). Site-level, multiplicity-adjusted zone tests did not support a difference at every depth, so the elevation pattern is reported descriptively.

For soil MeHg, we evaluated their data separately for dry (March) and wet (September) seasons. Dry-season topsoil MeHg averaged 0.248 ng g^–1^, 0.571 ng g^–1^, and 0.398 ng g^–1^ at lower, middle, and upper elevation, respectively; wet-season means were 0.512 ng g^–1^, 0.411 ng g^–1^, and 0.227 ng g^–1^ (Figure 2b), respectively. The increase from dry to wet season was the strongest at lower-elevation forest sites, whereas changes at middle- and upper-elevation sites were mixed. No elevation-zone contrast remained significant after site-level false-discovery-rate adjustment; individual site values and among-site variability are therefore shown in Figure 2b.

Among topsoils, % MeHg averaged 0.273%, 0.693%, and 0.423% at lower, middle, and upper elevation during the dry season, and 0.344%, 0.433%, and 0.235% during the wet season, respectively (Figure 2c; Tables S3–S5). These elevation-zone patterns did not remain significant after site-level false-discovery-rate adjustment.

### Factors Changing Soil Methylmercury over Time

The complete candidate set is shown in Table 1. The model containing ΔMoisture, plan curvature, and IHg had the lowest AICc, an Akaike weight of 0.500, adjusted *R* ^2^ = 0.758, and overall *p* = 0.0043. The parallel model containing elevation, plan curvature, and IHg had ΔAICc = 3.820, Akaike weight = 0.074, adjusted *R* ^2^ = 0.658, and *p* = 0.0141; the intercept-only model had ΔAICc = 3.045 and Akaike weight = 0.109. No single- or two-predictor model had an overall *p* < 0.05. The best-supported model retained the same coefficient directions with HC3 standard errors and in leave-one-site-out refits (Tables S7–S9). Substituting THg for IHg yielded a nearly identical site-level fit (adjusted *R* ^2^ = 0.757, overall *p* = 0.0044; Table S6).

**Table 1:** Candidate Set for Site-Level Models of Dry-to-Wet Seasonal Change in Topsoil MeHg (ΔMeHg; *n* = 11 Sites)^a^

model	predictors	*K*	AICc	ΔAICc	wi	adj. *R* ^2^	*p*
M10	ΔMoisture + plan curvature + IHg	5	6.441	0.000	0.500	0.758	0.0043
M0	intercept only	2	9.486	3.045	0.109	0.000	—
M2	elevation	3	9.938	3.496	0.087	0.190	0.1006
M11	elevation + plan curvature + IHg	5	10.261	3.820	0.074	0.658	0.0141
M1	ΔMoisture	3	11.231	4.790	0.046	0.089	0.1934
M7	elevation + plan curvature	4	11.354	4.912	0.043	0.356	0.0703
M5	ΔMoisture + plan curvature	4	11.681	5.240	0.036	0.337	0.0792
M4	IHg	3	11.898	5.457	0.033	0.032	0.2785
M9	plan curvature + IHg	4	11.993	5.551	0.031	0.318	0.0887
M3	plan curvature	3	12.403	5.962	0.025	–0.013	0.3760
M8	elevation + IHg	4	14.332	7.891	0.010	0.156	0.2078
M6	ΔMoisture + IHg	4	15.476	9.035	0.005	0.064	0.3150

a ΔMoisture and elevation were evaluated in parallel model families and were not fitted together because they were strongly correlated (*r* = −0.894). IHg = THg – MeHg; *K* includes regression coefficients and residual variance.

Elevation was strongly associated with ΔMoisture (*R* ^2^ = 0.80, *p* < 0.001; Figure 3a), so the two variables were evaluated in separate candidate-model families. ΔMoisture alone was weak (adjusted *R* ^2^ = 0.089, *p* = 0.193; Figure 3b), and elevation alone was also weak (adjusted *R* ^2^ = 0.190, *p* = 0.101). The model comparison supports a joint association of ΔMoisture, plan curvature, and IHg with ΔMeHg. Ridge sites tended to have lower topsoil % MeHg than valley sites, although site-level Mann–Whitney tests were not significant in the dry (*p* = 0.082) or wet season (*p* = 0.329; Figure 3c).

**Figure 3:**
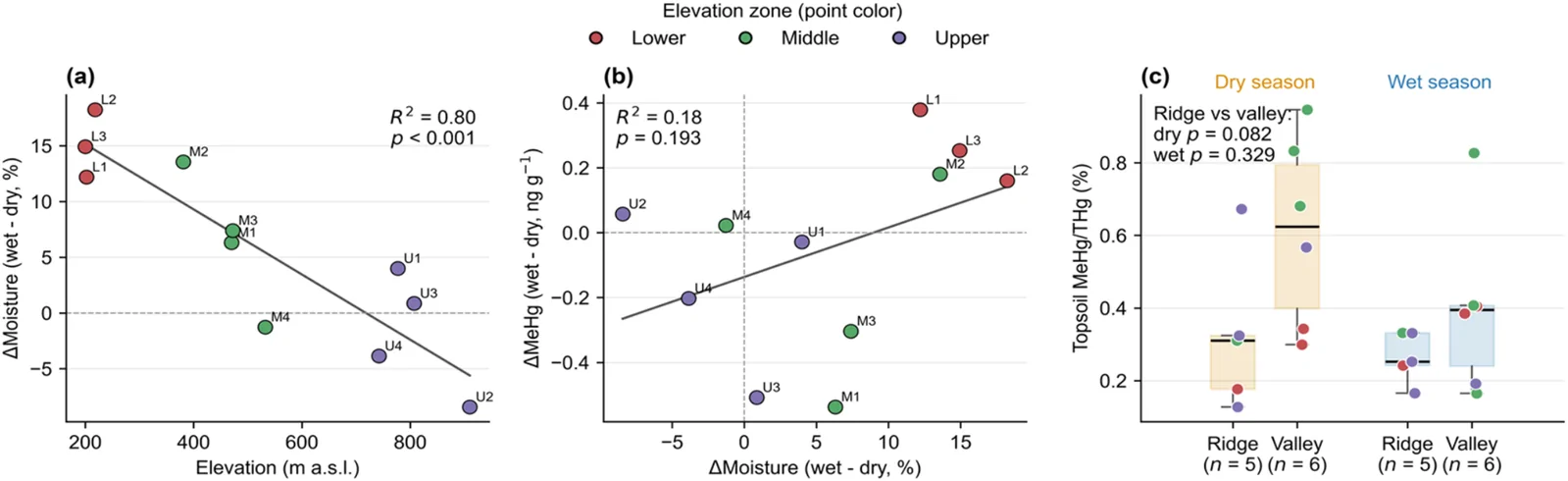
Site-level analyses (one independent value per site). (a) Seasonal moisture changes vs elevation; (b) seasonal MeHg change versus seasonal moisture change; points in panels (a, b) are colored by elevation zone (red, lower; green, middle; purple, upper). (c) Site-level boxplots of dry- and wet-season topsoil % MeHg for ridge (*n* = 5) and valley (*n* = 6) sites; boxes summarize site means and overlaid points show every site, colored by the elevation zone. The exact *p* values compare ridge and valley sites within each season using two-sided Mann–Whitney *U* tests.

In the replicate-level mixed model, the 191 observations were grouped by 11 sites and 66 physical cores to account for repeated depths; log­(IHg) was positively associated with MeHg (coefficient = 0.326, *p* < 0.001). TWI was positive in the full fit (coefficient = 0.227, *p* = 0.040) but was site-sensitive in leave-one-site-out analyses (*p* = 0.019–0.148). Depth contrasts were not significant in the reported model (4–10 versus 0–4 cm, *p* = 0.085; 10–20 versus 0–4 cm, *p* = 0.079), but depth inference was treated as descriptive because only 11 sites and 2 seasons were available; season, LOI, and instantaneous moisture were not significant. Marginal and conditional *R* ^2^ were 0.282 and 0.700, respectively (Tables S10 and S11). At the site level, no exploratory correlation of absolute topsoil MeHg with the measured predictors remained significant after within-season false-discovery-rate correction (Figure S2; Table S12).

## Discussion

Beyond direct deposition, throughfall and litterfall have been identified as important drivers of elevational THg gradients in soils.^39,40^ In regions with coniferous or mixed forests at middle elevations, THg inputs can follow the pattern: middle-elevation coniferous/mixed forest > lower-elevation broadleaf forest > upper-elevation alpine vegetation.^22^ Our study area lacks coniferous forest; broadleaf forest occurs at the lower elevation, while shrubland and grassland occur at middle and upper elevations (Figure S1). In a mixed model adjusted for depth and season, LOI was positively associated with THg within sites (standardized coefficient = 0.161, *p* < 0.001), while mean LOI showed no association with the among-site THg pattern (coefficient = −0.063, *p* = 0.580; SI, LOI–THg sensitivity analysis). Organic matter retention may therefore contribute to local THg variation, but it does not account for the watershed-scale site pattern.

The elevational pattern of topsoil THg in our study sites (Figure 2; Tables S3–S5) differs from some mountain systems with elevation ranges above 1000 m. For example, studies have found that higher atmospheric Hg deposition at higher elevations could lead to higher soil THg with increasing elevation.^21,39,41^ At our study sites, the elevation range was 200–910 m a.s.l. and vegetation changed from closed-canopy forest to shrubland and grassland above approximately 600 m a.s.l.; reduced canopy cover may lower vegetation-mediated atmospheric Hg inputs through foliar uptake and litterfall.^39,40^ Soil THg appeared to be quite comparable to those semiremote forest sites in the North America^40^ but lower than those influenced by point source Hg pollution such as those in Chinese mainland.^42^ Vegetation cover and elevation covary across this compressed gradient and cannot be statistically separated with our sampling design; vegetation is therefore treated as a qualitative correlate of the elevational THg pattern.^22^

MeHg and % MeHg varied with season and among individual sites (Figure 2b,c). The dry-to-wet increase was the strongest at lower-elevation sites, while upper-elevation sites generally decreased or changed little. The elevation-zone contrasts were not significant after site-level multiplicity correction, so these patterns are interpreted descriptively. Indeed, our elevational pattern was inconsistent with temperate systems with a higher elevational range in which researchers reported a linear increase in MeHg with elevation.^21,41^ Notably, neither study provided mechanistic explanations for the elevational gradient of MeHg.

Seasonal moisture change was associated with elevation and entered the best-supported model together with plan curvature and soil IHg (Figure 3a,b; Table 1). Along the elevational gradient, topsoil gained moisture from dry to wet season at lower- and middle-elevation sites but lost moisture at most upper-elevation sites. Greater saturation can restrict oxygen diffusion, create anoxic microsites, mobilize electron donors, and alter Fe, Mn, and sulfate reduction, all of which can affect Hg bioavailability and microbial Hg methylating activity.^43–45^ Rewetting can also stimulate organic matter turnover and microbial activity.^46^ However, redox potential, oxygen, sulfate, nitrate, Fe/Mn phases, dissolved organic matter, and *hgcAB* abundance were not measured, and direct measurements of these variables would help identify the processes underlying the seasonal pattern. Plot-level soil temperature was also unavailable; the two weather station series describe the climatic gradient but do not provide independent temperatures for the 11 plots. We believe that follow-up studies using genomic tools (e.g., targeting *hgcAB* genes)^47^ would be helpful in identifying the temporal and spatial hotspots of MeHg production in these terrestrial systems.

Plan curvature describes local convergence and drainage and may help explain spatial variation in soil moisture. Concave positions can retain water and solutes, although retention also depends on texture, mineralogy, soil structure, macropores, and organic matter.^48^ These soil properties were not measured, and the 30 m DEM cannot resolve all microrelief within the 5 m × 5 m plots. Plan curvature is therefore interpreted as a terrain proxy whose association requires testing with direct hydrological and soil-physical measurements. The nonsignificant ridge-valley contrasts and the site-sensitive TWI coefficient also limit inference from the topographic variables. Observed soil MeHg reflects the net balance of microbial methylation of Hg and demethylation of MeHg; no measured predictor showed a robust univariable site-level correlation with absolute topsoil MeHg after multiplicity adjustment, and resolving the opposing processes will require targeted incubation or molecular studies.^28^ Sites identified as valleys also behave similarly in water and solute retention.^48^ As related to Hg cycling, in a previous study at the upland-peatland interface, it was demonstrated that solutes such as nutrients from upland could stimulate net Hg methylation in the peatland soil porewater,^26^ suggesting the importance of microtopography on terrestrial Hg methylation processes that were largely overlooked in past studies. Future studies can combine the topographic features with biogeochemical Hg measurements in upland ecosystems.

IHg was used in the primary models because MeHg is one component of THg. This subtraction reduces direct component overlap but does not provide a direct measurement of the bioavailable Hg­(II). The positive IHg coefficient persisted in the site-level and replicate-level analyses, and substituting THg produced nearly identical results at both scales (site-level adjusted *R* ^2^ = 0.757, overall *p* = 0.0044; replicate-level standardized coefficient = 0.332, *p* < 0.001; Table S6). The association may partly reflect shared retention by soil organic matter; the positive within-site LOI–THg relationship and weak LOI–MeHg relationship indicate that Hg storage and net methylation can differ. Direct measurements of aqueous or ligand-bound Hg­(II), mineral phases, and demethylation are needed to resolve the substrate mechanism.^49^

In summary, seasonal topsoil MeHg change in this watershed was jointly associated with moisture changes, plan curvature, and soil IHg. Sites were the independent units for watershed-scale tests, and replicate-level models accounted for clustering by sites and repeated depths within physical cores. The study was limited to 11 sampling sites, one watershed, and one dry–wet seasonal transition, and several relevant mechanisms were not measured. The findings identify testable terrain-moisture hypotheses for future work across additional sites, watersheds, and years.^50^ Future work can investigate the microbial community compositions underlying the observed methylation patterns as well as the role of soil organic matter compositions in governing Hg­(II) speciation and bioavailability for microbial uptake and methylation in terrestrial ecosystems. Future studies should test whether valleys are hotspots of MeHg production during the dry season.

## Supplementary Material





## Data Availability

All data generated during this study are included in this article, its Supporting Information, and the separately supplied Data Set S1.

## Notes

Site attributes, seasonal soil measurements, model diagnostics and sensitivity analyses, site photographs, and supplementary maps (PDF)
Complete replicate-level raw data and site-level topographic attributes (XLSX)

## References

[1] Fitzgerald W. F., Engstrom D. R., Mason R. P., Nater E. A. (1998). The case for atmospheric
mercury contamination in remote areas. Environ.
Sci. Technol..

[2] Gilmour C.
C., Podar M., Bullock A. L., Graham A. M., Brown S. D., Somenahally A. C., Johs A., Hurt R. A., Bailey K. L., Elias D. A. (2013). Mercury methylation by novel microorganisms
from new environments. Environ. Sci. Technol..

[3] Jones D. S., Walker G. M., Johnson N. W., Mitchell C. P. J., Coleman
Wasik J. K., Bailey J. V. (2019). Molecular evidence for novel mercury
methylating microorganisms in sulfate-impacted lakes. ISME J..

[4] Peterson B. D., McDaniel E. A., Schmidt A. G., Lepak R. F., Janssen S. E., Tran P. Q., Marick R. A., Ogorek J. M., DeWild J. F., Krabbenhoft D. P., McMahon K. D. (2020). Mercury methylation
genes identified
across diverse anaerobic microbial guilds in a eutrophic sulfate-enriched
lake. Environ. Sci. Technol..

[5] Li Z., Wei T., He L., Qian H., Zhu Y.-G., Wang Y. (2025). Genomic potential
for mercury biotransformation in marine sediments across marginal
slope to hadal zone. Nat. Commun..

[6] Driscoll C. T., Mason R. P., Chan H. M., Jacob D. J., Pirrone N. (2013). Mercury as
a global pollutant: Sources, pathways, and effects. Environ. Sci. Technol..

[7] Sonke J. E., Angot H., Zhang Y., Poulain A., Björn E., Schartup A. (2023). Global change effects on biogeochemical mercury cycling. Ambio.

[8] Kronberg R.-M., Drott A., Jiskra M., Wiederhold J. G., Björn E., Skyllberg U. (2016). Forest harvest contribution to boreal
freshwater methyl mercury load. Global Biogeochem.
Cycles.

[9] Kronberg R.-M., Jiskra M., Wiederhold J. G., Björn E., Skyllberg U. (2016). Methyl mercury formation in hillslope soils of boreal
forests: The role of forest harvest and anaerobic microbes. Environ. Sci. Technol..

[10] Tsui M. T.-K., Liu S., Brasso R. L., Blum J. D., Kwon S. Y., Ulus Y., Nollet Y. H., Balogh S. J., Eggert S. L., Finlay J. C. (2019). Controls of methylmercury bioaccumulation in forest
floor food webs. Environ. Sci. Technol..

[11] Stinson I., Li H.-H., Tsui M. T.-K., Ku P., Ulus Y., Cheng Z., Lam H.-M. (2024). Tree foliage as
a net accumulator
of highly toxic methylmercury. Sci. Rep..

[12] Wang X., Yuan W., Lin C.-J., Zhang L., Zhang H., Feng X. (2019). Climate and vegetation as primary drivers for global mercury storage
in surface soil. Environ. Sci. Technol..

[13] Obrist D. (2012). Mercury distribution
across 14 U.S. forests. Part II: Patterns of methyl mercury concentrations
and areal mass of total and methyl mercury. Environ. Sci. Technol..

[14] Schelker J., Burns D. A., Weiler M., Laudon H. (2011). Hydrological mobilization
of mercury and dissolved organic carbon in a snow-dominated, forested
watershed: Conceptualization and modeling. J.
Geophys. Res.: Biogeosci..

[15] Jiskra M., Wiederhold J. G., Skyllberg U., Kronberg R. M., Kretzschmar R. (2017). Source tracing
of natural organic matter bound mercury in boreal forest runoff with
mercury stable isotopes. Environ. Sci.: Processes
Impacts.

[16] UNEP . UN Environment Programme, 2019. https://www.unep.org/resources/annual-report-2018 (accessed Mar 16, 2026).

[17] Zhang H., Wang W. X., Lin C. J., Feng X. B., Shi J. B., Jiang G. B., Larssen T. (2022). Decreasing
mercury levels in consumer
fish over the three decades of increasing mercury emissions in China. Eco-Environ. & Health.

[18] Li T., Mao H., Wang Z., Yu J. Z., Li S., Nie X., Herrmann H., Wang Y. (2023). Field evidence for Asian outflow
and fast depletion of total gaseous mercury in the polluted coastal
atmosphere. Environ. Sci. Technol..

[19] Chau K. C., Marafa L. M. (1999). Vegetation chronosequence
in Hong Kong: Soil properties
and successional development. Singapore J. Trop.
Geogr..

[20] Zhang F., Xu Z., Xu X., Liang L., Chen Z., Dong X., Luo K., Dinis F., Qiu G. (2022). Terrestrial mercury and methylmercury
bioaccumulation and trophic transfer in subtropical urban forest food
webs. Chemosphere.

[21] Gerson J. R., Driscoll C. T., Demers J. D., Sauer A. K., Blackwell B. D., Montesdeoca M. R., Shanley J. B., Ross D. S. (2017). Deposition of mercury
in forests across a montane elevation gradient: Elevational and seasonal
patterns in methylmercury inputs and production. J. Geophys. Res.: Biogeosci..

[22] Zeng S., Wang X., Yuan W., Luo J., Wang D. (2022). Mercury accumulation
and dynamics in montane forests along an elevation gradient in Southwest
China. J. Environ. Sci..

[23] Wang S., Xing D., Wei Z., Jia Y. (2013). Spatial and seasonal
variations in soil and river water mercury in a boreal forest, Changbai
Mountain, northeastern China. Geoderma.

[24] Gworek B., Dmuchowski W., Baczewska-Dąbrowska A. H. (2020). Mercury in the terrestrial
environment: A review. Environ. Sci. Eur..

[25] Du H., Wang X., Yuan W., Wu F., Jia L., Liu N., Lin C.-J., Gan J., Zeng F., Wang K., Feng X. (2023). Elevated mercury deposition,
accumulation, and migration in a karst
forest. Environ. Sci. Technol..

[26] Mitchell C. P. J., Branfireun B. A., Kolka R. K. (2008). Spatial characteristics
of net methylmercury production hot spots in peatlands. Environ. Sci. Technol..

[27] Tjerngren I., Meili M., Björn E., Skyllberg U. (2012). Eight boreal
wetlands as sources and sinks for methyl mercury in relation to soil
acidity, C/N ratio, and small-scale flooding. Environ. Sci. Technol..

[28] Wang S., Tsui M. T.-K., Li J., Pan K. (2024). Biogeochemical controls
on methylmercury distribution in a subtropical wetland ecosystem. Mar. Pollut. Bull..

[29] Ku P., Tsui M. T.-K., Nie X., Chen H., Hoang T. C., Blum J. D., Dahlgren R. A., Chow A. T. (2018). Origin, reactivity,
and bioavailability of mercury in wildfire ash. Environ. Sci. Technol..

[30] Tang W., Chu J., Zhong H. (2023). Alkaline extraction: An optimal approach for extracting
methylmercury from paddy soils. Sci. Total Environ..

[31] De
Reu J., Bourgeois J., Bats M., Zwertvaegher A., Gelorini V., De Smedt P., Chu W., Antrop M., De Maeyer P., Finke P., Van Meirvenne M., Verniers J., Crombé P. (2013). Application of the topographic position
index to heterogeneous landscapes. Geomorphology.

[32] Mattivi P., Franci F., Lambertini A., Bitelli G. (2019). TWI Computation: A
comparison of different open source GISs. Open
Geospatial Data Software Stand..

[33] Copernicus Data Space Ecosystem . Copernicus DEM - Global and European Digital Elevation Model [GLO-30 Dataset] 10.5270/ESA-c5d3d65.

[34] Conrad O., Bechtel B., Bock M., Dietrich H., Fischer E., Gerlitz L., Wehberg J., Wichmann V., Böhner J. (2015). System for
automated geoscientific analyses (SAGA) v. 2.1.4. Geosci. Model Dev..

[35] Guisan A., Weiss S. B., Weiss A. D. (1999). GLM versus CCA spatial modeling of
plant species distribution. Plant Ecol..

[36] Burnham K. P., Anderson D. R. (2001). Kullback-Leibler
information as a basis for strong
inference in ecological studies. Wildl. Res..

[37] Nakagawa S., Schielzeth H. (2013). A general
and simple method for obtaining R^2^ from generalized linear
mixed-effects models. Methods Ecol. Evol..

[38] Seabold, S. ; Perktold, J. In Statsmodels: Econometric and Statistical Modeling with Python, Proceedings of the 9th Python in Science Conference, 2010; pp 92–96 10.25080/Majora-92bf1922-011.

[39] Blackwell B. D., Driscoll C. T. (2015). Deposition of mercury
in forests along a montane elevation
gradient. Environ. Sci. Technol..

[40] Obrist D., Johnson D. W., Lindberg S. E., Luo Y., Hararuk O., Bracho R., Battles J. J., Dail D. B., Edmonds R. L., Monson R. K., Ollinger S. V., Pallardy S. G., Pregitzer K. S., Todd D. E. (2011). Mercury distribution across 14 U.S.
forests. Part I:
Spatial patterns of concentrations in biomass, litter, and soils. Environ. Sci. Technol..

[41] Zhang H., Yin R., Feng X., Sommar J., Anderson C. W. N., Sapkota A., Fu X., Larssen T. (2013). Atmospheric mercury inputs in montane soils increase
with elevation: evidence from mercury isotope signatures. Sci. Rep..

[42] Chen X., Ji H., Yang W., Zhu B., Ding H. (2016). Speciation and distribution
of mercury in soils around gold mines located upstream of Miyun Reservoir,
Beijing, China. J. Geochem. Explor..

[43] Poulin B. A., Aiken G. R., Nagy K. L., Manceau A., Krabbenhoft D. P., Ryan J. N. (2016). Mercury transformation
and release differs with depth
and time in a contaminated riparian soil during simulated flooding. Geochim. Cosmochim. Acta.

[44] Gfeller L., Weber A., Worms I., Slaveykova V. I., Mestrot A. (2021). Mercury mobility, colloid formation
and methylation
in a polluted Fluvisol as affected by manure application and flooding–draining
cycle. Biogeosciences.

[45] Coleman
Wasik J. K., Engstrom D. R., Mitchell C. P. J., Swain E. B., Monson B. A., Balogh S. J., Jeremiason J. D., Branfireun B. A., Kolka R. K., Almendinger J. E. (2015). The effects
of hydrologic fluctuation and sulfate regeneration on mercury cycling
in an experimental peatland. J. Geophys. Res.:
Biogeosci..

[46] Wang D., He N., Wang Q., Lü Y., Wang Q., Xu Z., Zhu J. (2016). Effects of temperature and moisture on soil organic matter decomposition
along elevation gradients on the Changbai Mountains, northeast China. Pedosphere.

[47] Liu Y. R., Johs A., Bi L., Lu X., Hu H. W., Sun D., He J. Z., Gu B. (2018). Unraveling
microbial communities
associated with methylmercury production in paddy soils. Environ. Sci. Technol..

[48] Fan Y., Clark M., Lawrence D. M., Swenson S., Band L. E., Brantley S. L., Brooks P. D., Dietrich W. E., Flores A., Grant G., Kirchner J. W., Mackay D. S., McDonnell J. J., Milly P. C. D., Sullivan P. L., Tague C., Ajami H., Chaney N., Hartmann A., Hazenberg P., McNamara J., Pelletier J., Perket J., Rouholahnejad-Freund E., Wagener T., Zeng X., Beighley E., Buzan J., Huang M., Livneh B., Mohanty B. P., Nijssen B., Safeeq M., Shen C., van Verseveld W., Volk J., Yamazaki D. (2019). Hillslope hydrology in global change
research and earth system modeling. Water Resour.
Res..

[49] Bloom N. S., Preus E., Katon J., Hiltner M. (2003). Selective extractions
to assess the biogeochemically relevant fractionation of inorganic
mercury in sediments and soils. Anal. Chim.
Acta.

[50] Li S., Chen Y., Wei W., Fang G., Duan W. (2024). The Increase
in extreme precipitation and its proportion over global land. J. Hydrol..

