# Supplementary material for: Microtopography and Seasonal Soil Moisture Govern Methylmercury Dynamics in Upland Soils of a Subtropical Coastal Montane Watershed
Source: ACS Omega. 2026 Sep 24;11(39):59754–61. doi: 10.1021/acsomega.6c06624 (PMC13647874; doi:10.1021/acsomega.6c06624)
Supplement: Supplementary file 1 [file ao6c06624_si_001.pdf]

Supporting Information (SI)

Microtopography and seasonal soil moisture govern methylmercury dynamics in upland soils of a subtropical coastal montane watershed

Xizi Yang [a], Martin Tsz-Ki Tsui [a,b,c,*], Zengchen Yu [a], Chenzi Yang [a], Mengwei Yuan [a], Shaoyi Wang [a], Ganxing Liu [a], Luming Yang [b], Lin Liu [b]

[a] *School of Life Sciences, The Chinese University of Hong Kong, Shatin, New Territories, Hong Kong SAR, China*

[b] *Department of Earth and Environmental Sciences, The Chinese University of Hong Kong, Shatin, New Territories, Hong Kong SAR, China*

[c] *State Key Laboratory of Marine Environmental Health, City University of Hong Kong, Kowloon Tong, Kowloon, Hong Kong SAR, China*

* Corresponding author (***M.T.-K. Tsui***); Address: Room G89 Science Centre, The Chinese University of Hong Kong, Shatin, New Territories, Hong Kong SAR, China; Phone: +852-3943 6123; E-mail: mtktsui@cuhk.edu.hk

Table S1 Per-site topographic attributes derived from the 30-m Copernicus DEM (SAGA/QGIS). The ridge/valley grouping used for Fig. 3c follows the natural TWI break.

Site	Zone	Elev. (m)	Slope	Aspect	Plan curv.	Profile curv.	TWI	TPI	Class
U2	upper	910	0.28	0.83	+0.0015	-0.0005	5.51	-0.36	ridge
U3	upper	807	0.22	2.06	-0.0008	-0.0010	8.10	-2.98	valley
U1	upper	777	0.18	0.90	+0.0008	+0.0001	5.52	+7.90	ridge
U4	upper	742	0.06	0.38	+0.0009	+0.0002	5.95	+5.34	ridge
M4	middle	532	0.21	2.20	+0.0033	-0.0008	4.83	+7.40	ridge
M3	middle	472	0.20	2.10	-0.0001	+0.0003	7.89	-1.85	valley
M1	middle	470	0.23	0.74	-0.0012	-0.0010	7.35	+1.13	valley
M2	middle	381	0.36	1.14	+0.0010	-0.0005	7.90	-1.87	valley
L2	lower	218	0.44	1.79	-0.0001	-0.0001	9.68	-6.77	valley
L1	lower	202	0.13	4.12	-0.0009	+0.0003	5.76	-2.14	ridge
L3	lower	200	0.19	1.73	+0.0003	-0.0016	7.87	-5.41	valley

Table S2 On-day and previous-day rainfall (Hong Kong Observatory, Tai Mo Shan station) for each sampling date.

Sampling date	On-day rainfall (mm)	Previous-day rainfall (mm)
31 Mar 2024	5.0	2.5
5 Apr 2024	0.5	0.5
10 Apr 2024	0.0	1.0
23 Aug 2024	0.0	0.5
28 Aug 2024	1.0	0.0
27 Sep 2024	0.5	0.0

Table S3 Mean ± SD of LOI, moisture, THg, MeHg, and %MeHg by site, depth, and season in the lower-elevation zone. Values summarize up to three cores; sample-size exceptions are listed in the note below.

			Dry season					Wet season				
Site	Elevation (m a.s.l.)	Soil depth (cm)	LOI (%)	Moisture (%)	THg (ng g⁻¹)	MeHg (ng g⁻¹)	%MeHg	LOI (%)	Moisture (%)	THg (ng g⁻¹)	MeHg (ng g⁻¹)	%MeHg
L1	202	0–4	7.4±0.0	13.3±0.6	158.2±17.9	0.28±0.06	0.18±0.03	11.7±2.0	25.5±4.5	274.0±26.1	0.66±0.21	0.24±0.08

Site	Elevation (m a.s.l.)	Soil depth (cm)	Dry season					Wet season				
			LOI (%)	Moisture (%)	THg (ng g⁻¹)	MeHg (ng g⁻¹)	%MeHg	LOI (%)	Moisture (%)	THg (ng g⁻¹)	MeHg (ng g⁻¹)	%MeHg
		4–10	7.0±0.4	13.4±1.9	180.1±14.2	0.28±0.06	0.15±0.03	8.6±1.1	21.4±2.0	292.4±21.7	0.40±0.01	0.14±0.01
		10–20	5.9±0.1	14.2±2.6	124.5±49.0	0.20±0.04	0.17±0.03	7.4±0.5	19.9±1.3	183.7±46.5	0.27±0.08	0.15±0.03
L2	218	0–4	13.0±2.6	17.9±3.1	90.8±23.1	0.27±0.06	0.30±0.07	12.7±2.6	36.2±6.4	110.8±4.6	0.43±0.10	0.38±0.09
		4–10	11.0±2.6	17.0±3.4	79.9±27.1	0.31±0.11	0.39±0.07	10.7±0.6	32.5±2.2	92.7±11.3	0.38±0.05	0.41±0.09
		10–20	10.6±1.5	16.7±3.0	70.0±13.4	0.24±0.04	0.35±0.03	10.2±0.8	37.2±1.7	81.3±12.2	0.40±0.11	0.52±0.21
L3	200	0–4	11.2±1.3	14.7±11.5	57.6±10.2	0.20±0.05	0.34±0.05	14.7±0.8	29.7±1.3	115.7±7.0	0.45±0.41	0.40±0.39
		4–10	7.4±0.6	19.3±2.1	35.7±25.0	0.17±0.08	0.83±0.77	11.5±1.1	26.4±1.6	99.5±26.3	0.20±0.07	0.22±0.11
		10–20	5.9±1.8	16.6±4.5	29.5±31.5	0.11±0.07	0.52±0.36	9.7±0.4	32.9±15.2	59.7±26.4	0.12±0.05	0.21±0.10

Table S4 Mean ± SD of LOI, moisture, THg, MeHg, and %MeHg by site, depth, and season in the middle-elevation zone. Values summarize up to three cores; sample-size exceptions are listed in the note below.

Site	Elevation (m a.s.l.)	Soil depth (cm)	Dry season					Wet season				
			LOI (%)	Moisture (%)	THg (ng g⁻¹)	MeHg (ng g⁻¹)	%MeHg	LOI (%)	Moisture (%)	THg (ng g⁻¹)	MeHg (ng g⁻¹)	%MeHg
M1	470	0–4	11.7±2.0	17.8 (*n* = 1)	85.3±5.3	0.71±0.14	0.83±0.11	9.6±0.2	24.1±2.7	111.8±52.5	0.18±0.06	0.16±0.03
		4–10	10.0±1.4	17.5±0.3	111.9±8.3	0.81±0.07	0.72±0.04	8.6±1.2	23.3±2.1	83.5±4.6	0.15±0.05	0.18±0.07
		10–20	7.0±0.2	16.4±0.7	68.4±10.2	0.48±0.18	0.71±0.30	6.3±0.9	18.6±0.6	63.1±21.8	0.14±0.04	0.23±0.06
M2	381	0–4	11.5±1.7	15.8±1.6	113.5±10.0	0.78±0.17	0.68±0.10	13.0±2.3	29.4±3.1	115.8±14.9	0.96±0.33	0.83±0.28
		4–10	7.8±1.0	13.6±1.0	110.8±4.3	0.38±0.12	0.34±0.10	9.3±0.6	22.1±2.4	101.5±19.0	0.75±0.21	0.74±0.14
		10–20	7.4±0.3	13.7±0.7	88.0±11.9	0.41±0.10	0.47±0.11	9.6±1.0	22.9±3.0	94.9±2.8	1.01±0.33	1.06±0.37
M3	472	0–4	12.6±1.0	20.0±1.2	70.7±12.5	0.63±0.28	0.95±0.56	14.0±2.6	27.4±3.0	80.8±9.7	0.33±0.05	0.41±0.01
		4–10	9.6±0.3	19.1±0.5	53.4±5.3	0.29±0.03	0.55±0.06	10.0±1.6	22.6±1.0	67.2±9.5	0.28±0.08	0.42±0.09
		10–20	8.5±0.4	18.6±0.5	32.4±4.4	0.19±0.08	0.57±0.22	8.3±0.4	20.8±0.3	37.9±4.9	0.15±0.02	0.41±0.03
M4	532	0–4	9.0±2.4	17.6±2.2	48.1±11.8	0.16±0.10	0.31±0.19	8.6±3.5	16.4±4.9	55.2±15.5	0.18±0.05	0.33±0.08
		4–10	8.3±0.8	16.4±2.4	53.7±2.0	0.20±0.07	0.37±0.15	9.4±0.3	21.4±2.1	62.9±4.8	0.20±0.08	0.32±0.11
		10–20	7.8±0.6	15.8±1.3	52.2±10.8	0.24±0.09	0.47±0.23	9.2±0.8	19.8±1.2	66.1±4.7	0.25±0.12	0.38±0.17

Table S5 Mean ± SD of LOI, moisture, THg, MeHg, and %MeHg by site, depth, and season in the upper-elevation zone. Values summarize up to three cores; sample-size exceptions are listed in the note below.

Site	Elevation (m a.s.l.)	Soil depth (cm)	Dry season					Wet season				
			LOI (%)	Moisture (%)	THg (ng g⁻¹)	MeHg (ng g⁻¹)	%MeHg	LOI (%)	Moisture (%)	THg (ng g⁻¹)	MeHg (ng g⁻¹)	%MeHg
U1	777	0–4	17.6±0.7	25.0±3.1	78.9±11.1	0.24±0.16	0.32±0.24	17.9±1.0	28.9±7.8	84.9±4.8	0.21±0.05	0.25±0.08
		4–10	14.7±1.7	23.0±2.0	77.9±17.7	0.28±0.15	0.35±0.14	13.1±0.5	24.8±3.4	72.7±14.2	0.26±0.12	0.38±0.22
		10–20	12.6±1.9	24.3±5.9	49.4±24.6	0.17±0.06	0.36±0.09	10.7±3.6	20.5±3.3	53.2±14.5	0.52±0.21	1.10±0.78
U2	910	0–4	22.1±8.7	38.2±4.9	119.1±26.9	0.14±0.09	0.13±0.09	14.8±0.2	29.8±1.5	119.4±2.3	0.20±0.05	0.17±0.03
		4–10	14.3±3.5	29.6±3.9	138.9±83.8	0.09±0.07	0.09±0.09	11.3±0.4	25.3±1.1	110.1±18.2	0.14±0.03	0.13±0.02
		10–20	10.1±0.3	25.3±1.3	94.1±27.3	0.07±0.03	0.08±0.03	10.6±0.3	23.7±0.3	99.7±19.0	0.12±0.03	0.12±0.02
U3	807	0–4	20.8±8.8	33.3±5.6	132.2±72.8	0.70±0.32	0.57±0.26	14.5±2.2	34.1±1.1	113.6±35.7	0.19±0.05	0.19±0.11
		4–10	9.2±1.1	23.9±2.7	75.3±16.2	0.27±0.09	0.37±0.12	7.9±0.6	24.5±1.6	80.7±18.5	0.13±0.03	0.17±0.07

Site	Elevation (m a.s.l.)	Soil depth (cm)	Dry season LOI (%)	Dry season Moisture (%)	Dry season THg (ng g^{-1})	Dry season MeHg (ng g^{-1})	Dry season %MeHg	Wet season LOI (%)	Wet season Moisture (%)	Wet season THg (ng g^{-1})	Wet season MeHg (ng g^{-1})	Wet season %MeHg
		10–20	6.4±0.3	19.5±0.3	37.8±6.6	0.14±0.01	0.38±0.06	7.0±1.0	22.3±2.4	51.7±15.9	0.10±0.02	0.19±0.03
U4	742	0–4	11.2±2.9	21.2±0.9	74.5±4.5	0.51±0.24	0.67±0.28	9.9±1.7	17.3 (*n* = 1)	91.3±8.8	0.31±0.12	0.33±0.11
		4–10	9.2±0.9	20.5±1.5	75.1±6.1	0.31±0.11	0.41±0.11	7.2±0.5	22.3±11.5	73.1±6.3	0.16±0.07	0.23±0.12
		10–20	8.2±0.4	18.4±1.6	61.6±6.7	0.20±0.11	0.34±0.21	8.4±0.4	25.9±15.0	78.8±20.5	0.09±0.01	0.11±0.01

Note for Tables S3–S5. Values are means ± SD of three cores unless otherwise stated. Moisture used n = 1 (SD not estimable) for M1 dry-season 0–4 cm and U4 wet-season 0–4 cm, and n = 2 for M1 wet-season 10–20 cm and U3 dry-season 4–10 cm. LOI used n = 2 for U3 wet-season 0–4 cm. Across all 198 observations, the mixed model used 191 complete cases because six moisture values and one LOI value were unavailable; the LOI–THg model used 197 observations because one LOI value was unavailable. The two below-detection-limit MeHg measurements were retained as measured. %MeHg was calculated for each core from its own season-specific MeHg and THg and then averaged. Triplicate cores describe within-plot variability; sites are the independent units for watershed-scale inference.

Table S6 Sensitivity of the site-level seasonal-change and replicate-level mixed models to replacing IHg with THg as the substrate predictor.

Analysis	Substrate predictor	Model fit	Substrate coefficient	*p*
Site-level seasonal change	IHg (primary)	adjusted R^2 = 0.758; overall *p* = 0.0043	0.0051	0.0062
Site-level seasonal change	THg (sensitivity)	adjusted R^2 = 0.757; overall *p* = 0.0044	0.0051	0.0063
Replicate-level mixed model	log(IHg), standardized	AIC = 320.834	0.3260	<0.001
Replicate-level mixed model	log(THg), standardized	AIC = 319.899	0.3319	<0.001

Substituting THg for IHg gave nearly identical results at both scales: the site-level model had adjusted R^2 = 0.757 and overall p = 0.0044, and the replicate-level mixed model had a standardized log(THg) coefficient of 0.332 ($p < 0.001$). Site-level substrate coefficients are on the original concentration scale; mixed-model substrate predictors were log-transformed and standardized. The *p* column refers to the substrate coefficient; overall site-level *p* values are shown under Model fit.

Table S7 Coefficients for the best-supported seasonal-change model; HC3 standard errors address heteroskedasticity.

Term	Estimate	SE	*p*	HC3 SE	HC3 *p*
Intercept	-0.8174	0.1598	0.0014	0.1245	0.0003
dMoisture	0.0246	0.0062	0.0055	0.0085	0.0229
PlanCurv	227.8412	46.5093	0.0018	79.7088	0.0244
IHg_mean	0.0051	0.0013	0.0062	0.0010	0.0015

Table S8 Variance-inflation factors for the primary site-level model.

Term	VIF
dMoisture	1.240
PlanCurv	1.618
IHg_mean	1.382

Table S9 Leave-one-site-out sensitivity of the primary seasonal-change model.

Site omitted	adj. R^2	Model *p*	β ΔMoisture	β plan curvature	β IHg
L1	0.677	0.0200	0.0244	225.691	0.0048
L2	0.739	0.0107	0.0231	227.751	0.0051
L3	0.803	0.0047	0.0207	224.740	0.0055
M1	0.679	0.0196	0.0235	202.496	0.0047
M2	0.785	0.0061	0.0281	251.502	0.0054
M3	0.726	0.0124	0.0246	225.567	0.0050
M4	0.835	0.0028	0.0254	293.035	0.0050
U1	0.764	0.0080	0.0247	228.367	0.0052
U2	0.803	0.0047	0.0297	214.582	0.0044

Site omitted	adj. R^2	Model p	β ΔMoisture	β plan curvature	β IHg
U3	0.740	0.0106	0.0213	201.539	0.0050
U4	0.756	0.0088	0.0263	231.544	0.0052

Model diagnostics and sensitivity. M4 had the largest Cook's distance (0.915), and L1 had the highest leverage (0.807). Leave-one-site-out refits retained all coefficient directions and overall model p values of 0.0028–0.0200. Because topsoil moisture was available for one core at M1 in the dry season and one core at U4 in the wet season, excluding both sites retained all coefficient directions (adjusted $R^2 = 0.648$; overall $p = 0.0425$).

Table S10 Linear mixed-effects model of log(MeHg), with random intercepts for site and physical core (each core was unique within one site and season; 191 complete observations; 66 cores; 11 sites; maximum likelihood). Continuous predictors are standardized.

Fixed effect	Estimate	SE	z	p
Intercept	-1.2708	0.1339	-9.492	<0.001
Depth: 4–10 cm	-0.1439	0.0835	-1.724	0.0846
Depth: 10–20 cm	-0.1669	0.0950	-1.756	0.0791
Season: wet	-0.0889	0.1217	-0.730	0.4652
log(IHg), standardized	0.3260	0.0646	5.050	<0.001
LOI, standardized	0.0542	0.0532	1.018	0.3088
Moisture, standardized	-0.0649	0.0599	-1.083	0.2790
TWI, standardized	0.2272	0.1107	2.052	0.0401

Site random-intercept variance = 0.097; core random-intercept variance = 0.140; residual variance = 0.170; marginal $R^2 = 0.282$; conditional $R^2 = 0.700$. Residual Shapiro–Wilk W = 0.969, $p < 0.001$; therefore the mixed model is treated as a supporting analysis and interpreted alongside leave-one-site-out and substrate-sensitivity results. With only 11 sites and two seasons, depth and site-level TWI terms are not used as primary evidence.

Table S11 Leave-one-site-out sensitivity of the mixed model described in Table S10.

Site omitted	β TWI	TWI p	β log(IHg)	IHg p
L1	0.2197	0.0624	0.3293	<0.001
L2	0.2788	0.0439	0.3362	<0.001
L3	0.2397	0.0531	0.3704	<0.001
M1	0.2177	0.0582	0.2866	<0.001
M2	0.1777	0.0562	0.3032	<0.001
M3	0.2110	0.0714	0.3091	<0.001
M4	0.2668	0.0340	0.2855	<0.001
U1	0.2602	0.0314	0.3515	<0.001
U2	0.1316	0.1481	0.3505	<0.001
U3	0.2555	0.0190	0.2908	<0.001
U4	0.2335	0.0542	0.3585	<0.001

Table S12 Site-level exploratory Spearman correlations for topsoil MeHg. q values are Benjamini–Hochberg adjusted within season.

Season	Predictor	n	ρ	p	q
dry	LOI	11	-0.009	0.9788	0.9788
dry	Moisture	11	-0.127	0.7092	0.8274
dry	Elevation	11	-0.155	0.6500	0.8274
dry	IHg	11	0.291	0.3855	0.8274
dry	TWI	11	0.618	0.0426	0.2985
dry	TPI	11	-0.155	0.6500	0.8274
dry	PlanCurv	11	-0.491	0.1252	0.4382
wet	LOI	11	0.227	0.5015	0.5851
wet	Moisture	11	0.264	0.4334	0.5851
wet	Elevation	11	-0.645	0.0320	0.2237
wet	IHg	11	0.409	0.2115	0.4531
wet	TWI	11	0.373	0.2589	0.4531
wet	TPI	11	-0.536	0.0890	0.3113
wet	PlanCurv	11	-0.009	0.9788	0.9788

LOI–THg sensitivity analysis. A separate mixed model of log(THg), with random intercepts for site and physical core and with depth and season as covariates, found a positive association with standardized LOI (coefficient = 0.157, $p < 0.001$; $n = 197$ observations from 66 cores across 11 sites; one LOI value was unavailable). The overall LOI × depth interaction was not significant (likelihood-ratio $\chi^2(2) = 4.40$, $p = 0.111$); therefore, the depth-specific fits (0–4 cm: 0.157, $p < 0.001$; 4–10 cm: 0.484, $p < 0.001$; 10–20 cm: 0.129, $p = 0.016$) are descriptive and do not demonstrate

different slopes among depths. A within/between-site decomposition indicated a positive within-site association (coefficient = 0.161, $p < 0.001$), whereas the among-site mean-LOI term was not significant (coefficient = −0.063, $p = 0.580$). LOI therefore covaried with THg locally but did not explain broad among-site THg differences.

Fig. S1 Vegetation at sampling sites in Shing Mun and Tai Mo Shan Country Parks, Hong Kong SAR, China. Photographs by X. Yang.

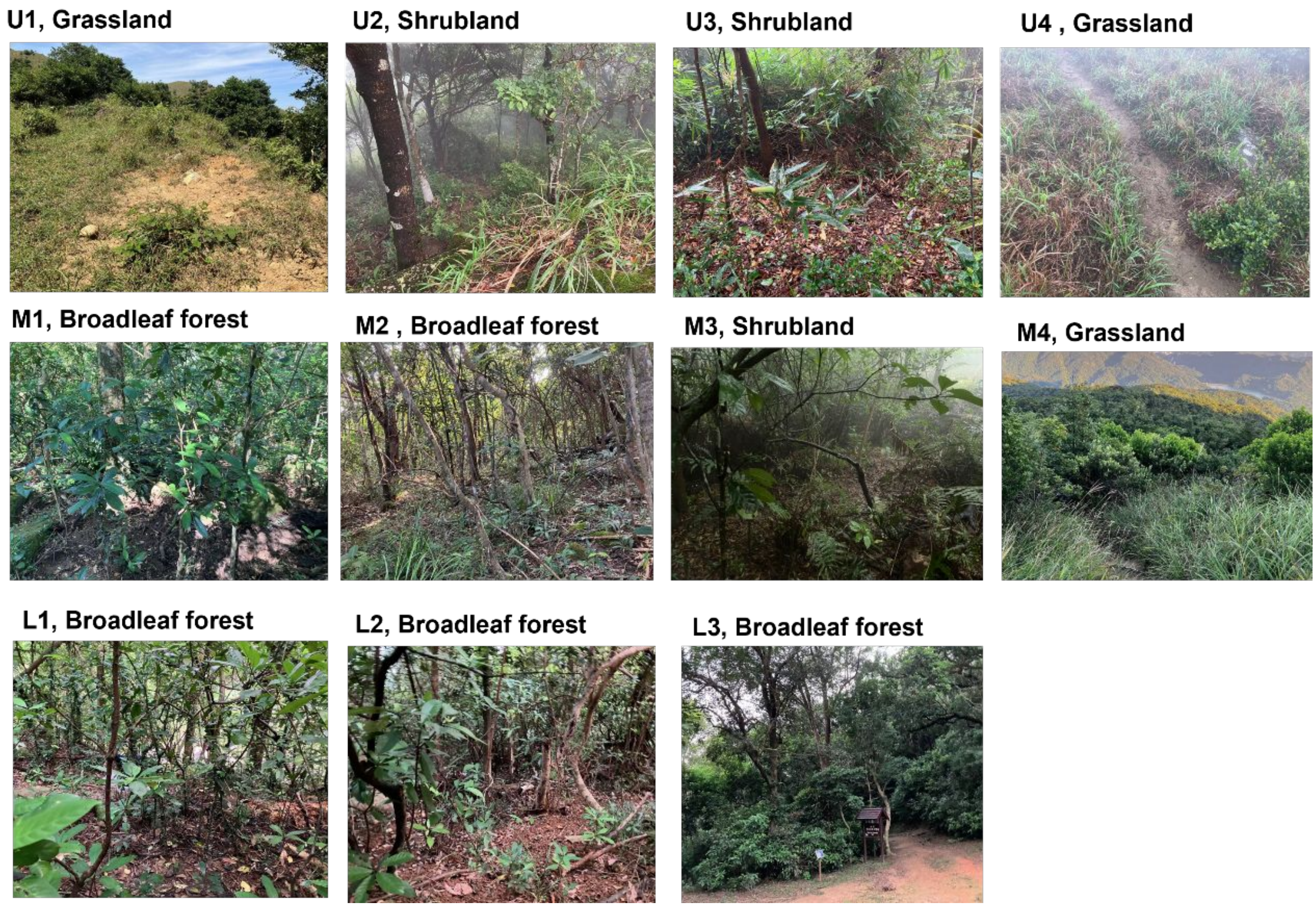

Fig. S2 Site-mean topsoil (0–4 cm) MeHg versus measured explanatory variables by season (n = 11 sites per season). ρ is Spearman's coefficient; p is unadjusted and q is Benjamini–Hochberg adjusted within season.

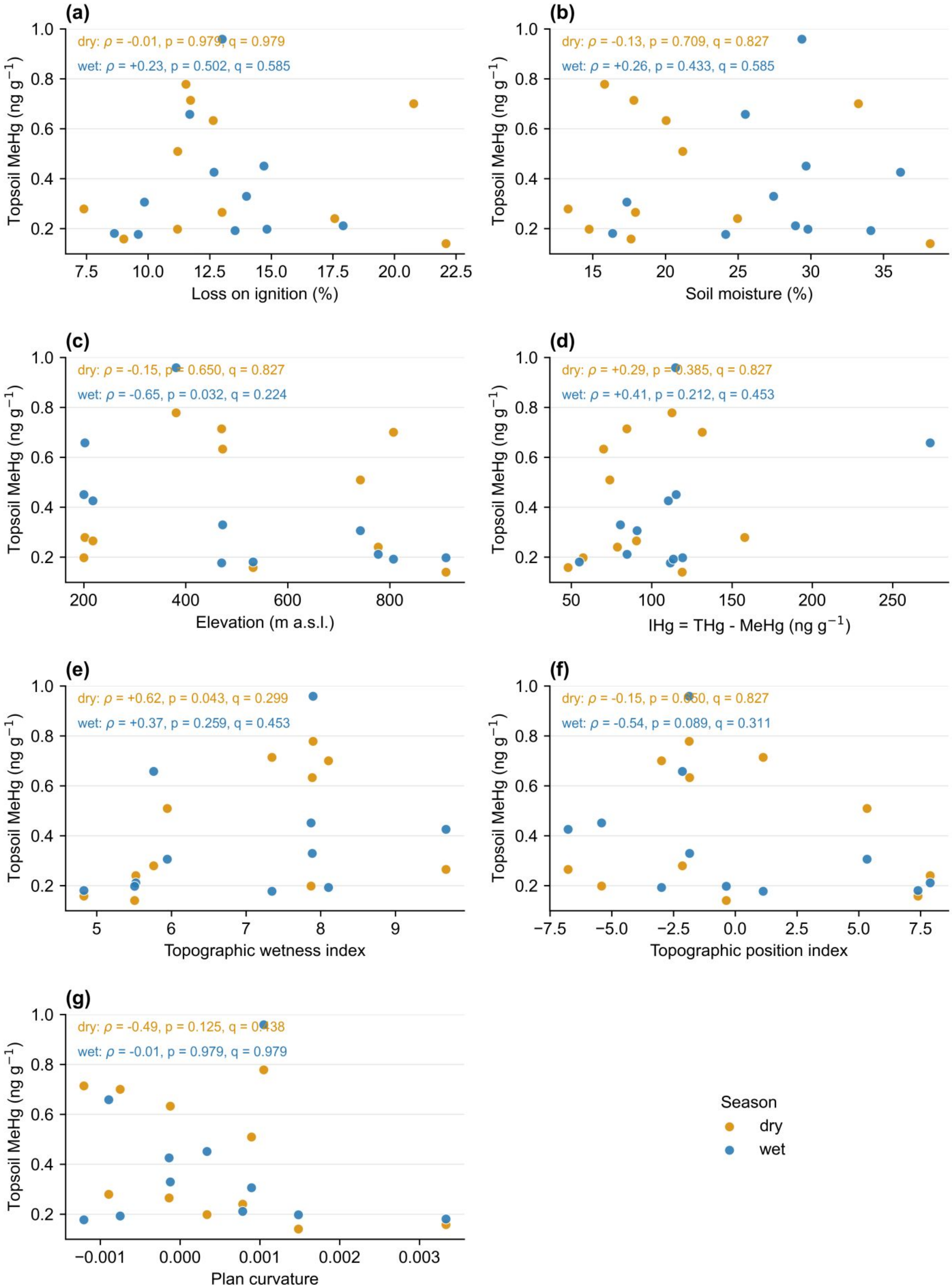

Figure S3 **Sampling sites and DEM-derived stream network**

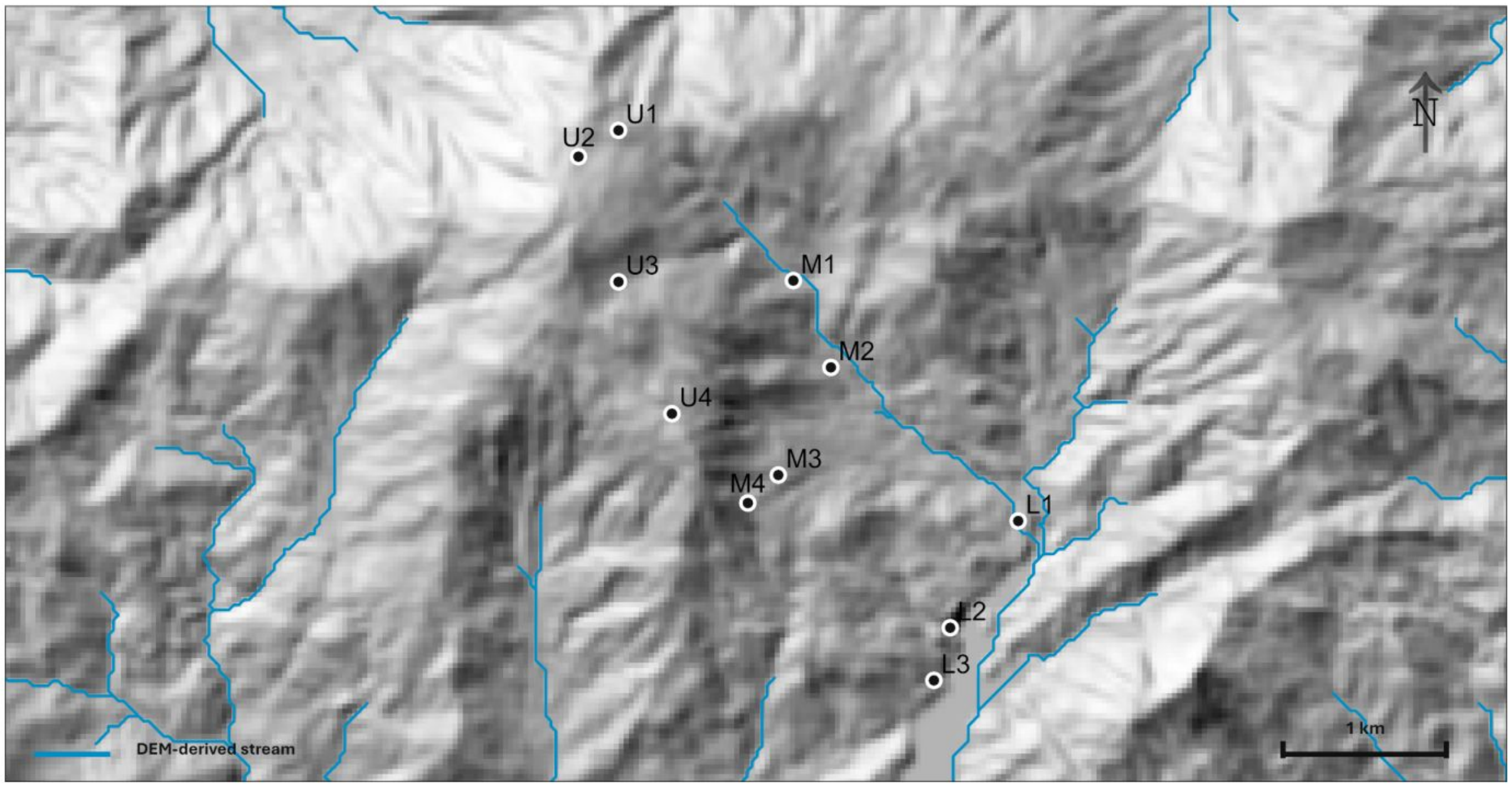

Fig. S3 Locations of the 11 field sampling sites and the DEM-derived stream network in the Tai Mo Shan study area. Site elevations used in the analyses were measured in the field. The Copernicus GLO-30 DEM was used to represent terrain and derive the stream network from a filled DEM using D8 flow accumulation.

Dataset S1 Complete replicate-level raw dataset (198 records) of LOI, soil moisture, THg, MeHg, and per-site topographic attributes. The file is supplied separately as SI_DatasetS1_raw.xlsx.
